# Comparing the Prevalence and Characteristics of Chest Pain in Children and Adolescents Pre- and Post-COVID-19: A Retrospective Study

**DOI:** 10.7759/cureus.71407

**Published:** 2024-10-14

**Authors:** Iyad Alammouri, Aya E Odeh, Farah A Alkhaled, Razan M Al-zoubi, Yaqin M Alzagareet

**Affiliations:** 1 Pediatrics, School of Medicine, The University of Jordan, Amman, JOR

**Keywords:** cardiac problem, chest pain, covid-19, pediatrics, post-covid era, pre-covid-19

## Abstract

Background: Chest pain is a common complaint among pediatric patients, often leading to visits to Emergency Departments or outpatient clinics. While most cases are benign, timely diagnosis is essential to prevent fatalities in those with serious conditions. The COVID-19 pandemic has shifted healthcare dynamics, necessitating an understanding of its impact on pediatric health, including potential complications such as chest pain, fever, cough, shortness of breath, sore throat, and headache. This study aims to explore the prevalence, characteristics, and potential association between COVID-19 and chest pain in children during two time periods: 2019 (before the COVID-19 pandemic) and 2021 (the full year during the pandemic).

Methodology: Data were collected from medical records and telephone interviews with pediatric patients presenting with chest pain at the University of Jordan Hospital. The study included a sample size of 3294 patients with selection criteria based on presenting symptoms and COVID-19 status. Data collection occurred from 2019 and 2021, and demographic information (age, gender, weight), medical history (perinatal and family history), COVID-19 status (vaccination, infection history), and details about chest pain (frequency, onset) were documented. Statistical analyses were performed to evaluate differences between the two time periods using IBM SPSS Statistics for Windows, Version 28 (Released 2021; IBM Corp., Armonk, New York, United States).

Results: The results indicate a significant increase in the number of patients presenting with chest pain in 2021 compared to 2019 (P value = .0157). The mean age of patients was 11 years, predominantly male. There was a notable increase in the percentage of patients with comorbidities in 2021. Echocardiography results were normal in approximately 84% of cases across both years, with no significant differences in ECG outcomes. Palpitations remained the most common associated symptom. 4.6 % of patients in 2021 were confirmed to have COVID-19, most of whom were diagnosed with muscular causes of chest pain. Notably, none of the patients had received the COVID-19 vaccine.

Conclusions: This study highlights a marked increase in pediatric patients presenting with chest pain during the pandemic, although characteristics and underlying causes remained consistent between years. The rise in cases may be attributed to heightened awareness rather than a direct link to COVID-19. Recommendations for clinical practice include careful evaluation of chest pain in pediatric patients during the pandemic, considering psychosocial factors and the broader impact of COVID-19 on health-seeking behaviors. Future research should investigate the long-term effects of COVID-19 on pediatric populations and explore the psychological implications on health service utilization. This study underscores the need for ongoing assessment of pediatric healthcare practices amid evolving pandemic conditions.

## Introduction

The incidence of chest pain in children and adolescents is high, making it the second most frequent complaint leading to referrals to pediatric cardiologists, murmurs being the comments [1]. While the majority of cases are not associated with underlying cardiac pathology, various conditions can contribute to chest pain in this population, including musculoskeletal disorders, respiratory issues, gastrointestinal problems, and anxiety-related disorders. Clinicians remain concerned about the potential for missing serious cardiac diagnoses, which could lead to sudden cardiac death [2].

In recent years, the COVID-19 pandemic has dominated research attention; however, studies focusing on long-term recovery from SARS-CoV-2 infection in children have been limited, often characterized by small sample sizes, lack of control groups, and insufficient exploration of specific symptoms related to chest pain [3]. Although the prognosis for most pediatric patients with COVID-19 is favorable, those with significant medical abnormalities require rapid recognition and appropriate treatment to ensure full recovery. Symptoms such as persistent chest pain, chronic headaches, fatigue, disrupted sleep, attention deficits, myalgia, arthralgia, abdominal discomfort, diarrhea, heart palpitations, and skin lesions may indicate long COVID, a condition defined by the persistence of symptoms for more than four weeks following acute infection [4].

Recent studies suggest that COVID-19 may have implications for the cardiovascular system, revealing statistically significant variations in echocardiographic measurements between study and control groups [5]. In pediatric populations, these cardiovascular effects could lead to potential long-term impacts, including myocardial inflammation, altered cardiac function, and increased risk of arrhythmias, highlighting the need for careful monitoring and evaluation.

Given the growing body of literature on chest pain with COVID-19 in adults, there is a need to investigate the prevalence of chest pain in children during two time periods: 2019 (before the pandemic) and 2021 (during the pandemic). This study aims to examine the impact of COVID-19 on clinic visits, the characteristics, and etiology of chest pain in children, including whether it is cardiac in origin. Given the scarcity of data on chest pain in children with COVID-19, this study will contribute to the knowledge gap in this area.

The study's findings may have important implications for clinical practice, helping healthcare providers to more effectively identify and treat chest pain in pediatric patients. By comprehending the characteristics and potential origins of chest pain within the context of COVID-19, clinicians can enhance diagnostic precision and create personalized treatment plans. Regarding policy development, the outcomes could guide the creation of guidelines for managing pediatric patients recovering from COVID-19, ensuring that healthcare systems are prepared to address this emerging issue efficiently. Moreover, the study could set the stage for future research by pinpointing crucial areas for further exploration, such as the long-term cardiovascular impacts of COVID-19 in children and the necessity for larger, controlled studies to establish causation and optimal management strategies.

## Materials and methods

This retrospective, cross-sectional study aimed to investigate the prevalence and characteristics of chest pain in pediatric patients (under 18 years old) at the University of Jordan Hospital in Amman, Jordan. The cross-sectional aspect refers to a specific time point within the retrospective data collection. Data was collected from medical records of pediatric patients diagnosed with chest pain, with a focus on those who met predefined inclusion criteria. Exclusion criteria included children with pre-existing cardiac conditions and those with incomplete records. The study team subsequently contacted participants by telephone to gather additional information, with both parents and children serving as respondents. Follow-up questions were standardized to ensure consistency.

The collected data included demographic information (age, gender, weight), medical history (perinatal and family history), and COVID-19 status, specifically confirming infection through positive PCR tests or clinical diagnoses. Vaccination status and the timeline of any COVID-19 infections in relation to chest pain episodes were also recorded. Additionally, the study documented the number of chest pain episodes and whether they occurred after a COVID-19 infection.

Investigations such as echocardiography and ECG were ordered based on clinical indications, and their results were recorded. Data analysis was performed using IBM SPSS Statistics for Windows, Version 28 (Released 2021; IBM Corp., Armonk, New York, United States), applying specific statistical methods, including descriptive statistics and significance testing.

Verbal consent from the parents of participants was obtained prior to the study, and they were informed of their right to refuse or withdraw from participation at any time. Participants were informed of the study's aim and potential benefits, and that the collected information would be treated with utmost confidentiality and privacy, with only the research team having access to the data. The study was conducted with strict adherence to confidentiality, with data anonymized and stored in encrypted databases to ensure compliance with data protection standards. Additionally, the study protocol was reviewed and approved by the institutional review board (IRB) of The University of Jordan, ensuring ethical conduct.

While the retrospective design allows for the exploration of existing data, it is important to acknowledge potential limitations, including reliance on medical records and self-reported information, which may introduce biases such as selection bias, recall bias, and confirmation bias in interpreting chest pain in the context of COVID-19.

## Results

A total of 3294 patients visited the clinic in 2019 and 2021, with 1762 and 1532 patients, respectively. Out of these patients, 283 had chest pain, with 132 patients in 2019 and 151 in 2021. Analyzing the data using the SPSS program for the statistical analysis and chi-square tests for the calculation of the P-values revealed a significant increase in the number of patients presenting with chest pain in 2021 compared to 2019 (P value = .0157). These patients with chest pain were selected as the sample for the study. Out of the patients presenting with chest pain in 2021, 4.6% were confirmed to have had COVID-19. This represents a relatively small portion of the total sample.

**Figure 1:**
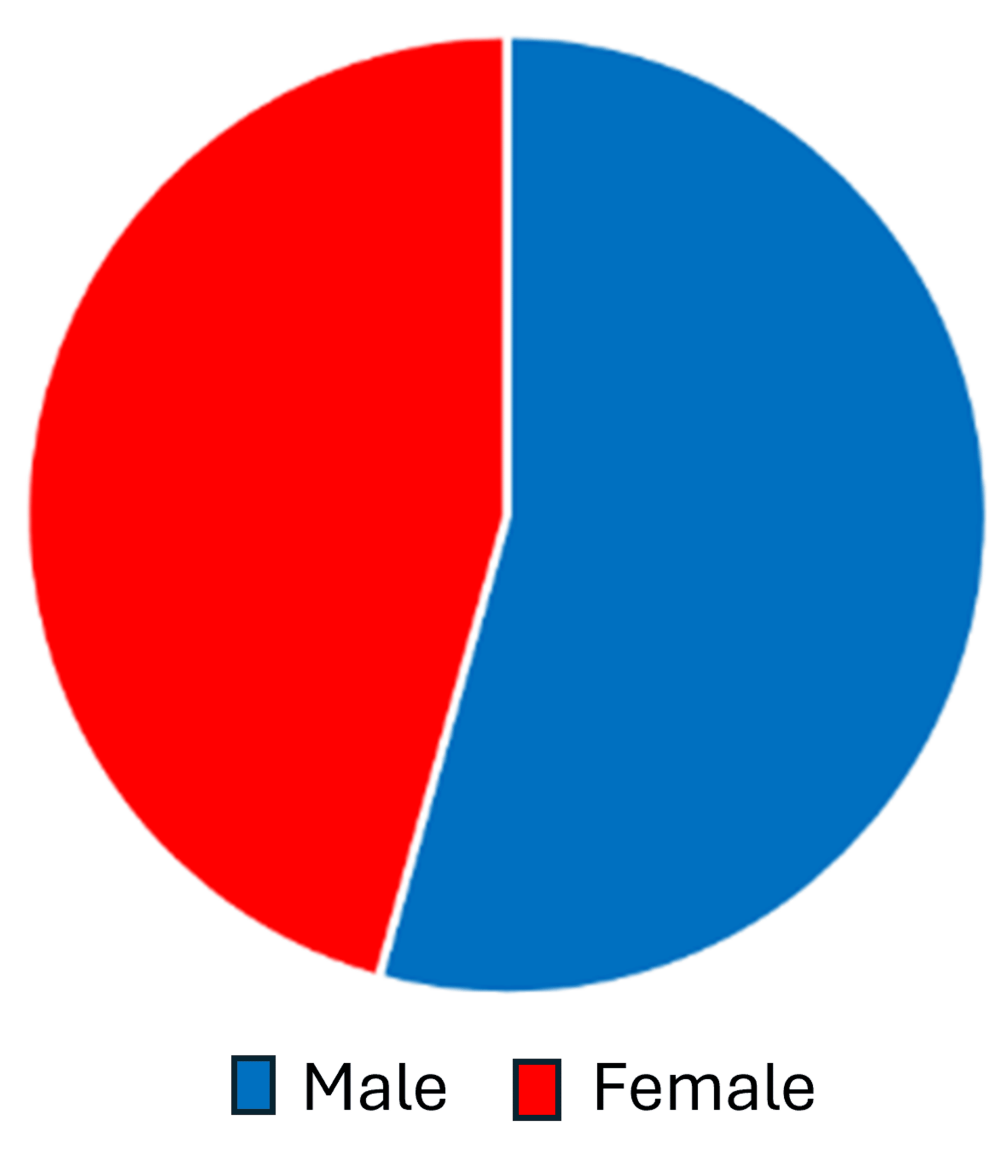
Gender distribution The overall sample was predominantly male (57.6% male and 42.4% female). This gender distribution is likely similar among the COVID-19-positive patients.

**Figure 2:**
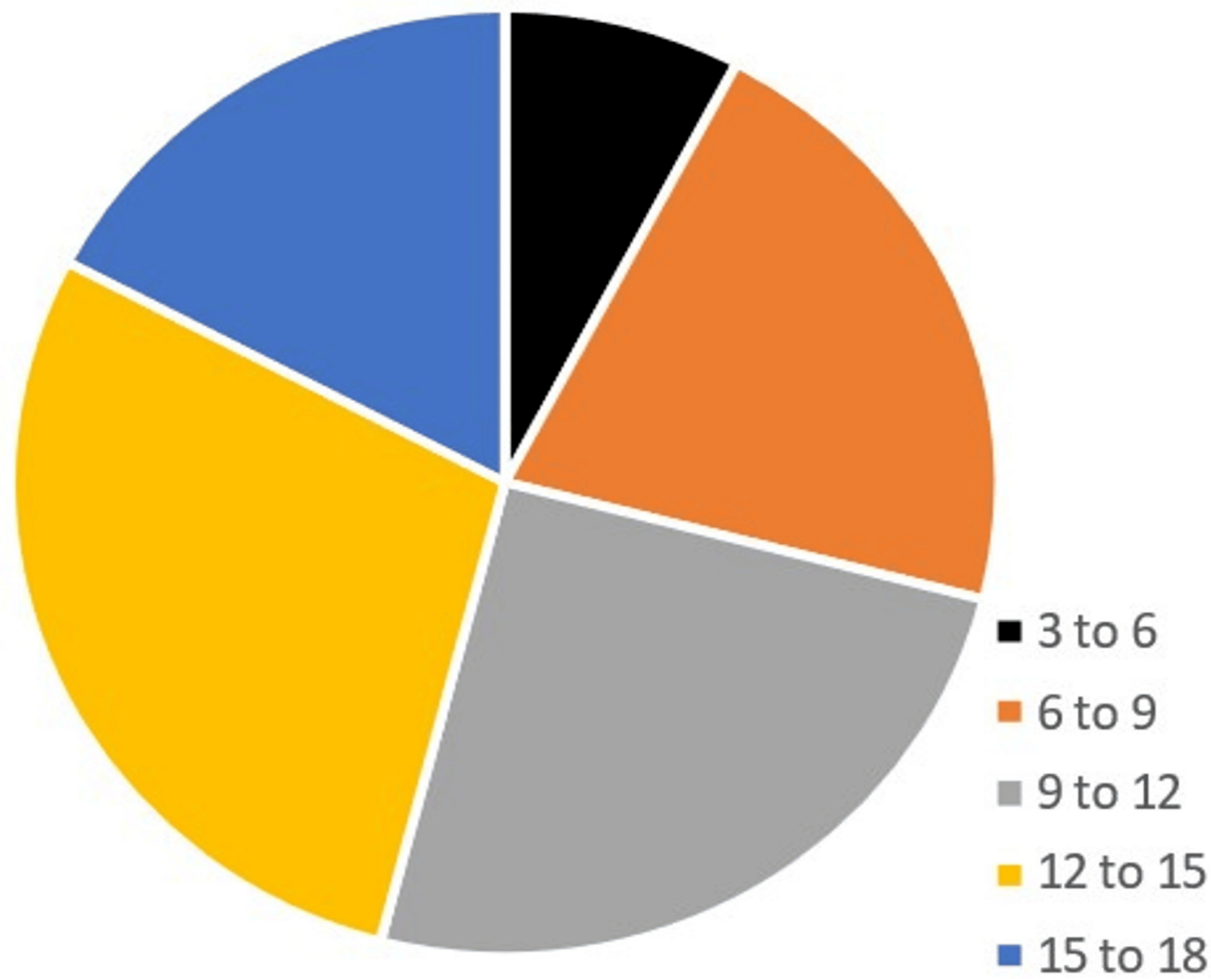
Age group distribution with age presented in years The mean age of patients across both years (2019 and 2021) was 11 years. It is reasonable to assume that the age distribution of COVID-19-positive patients is similar.

Patients' ages varied between 3 and 18 years, while the mean age (in years) was 11 in both 2019 and 2021. In 2019, 81 males visited the clinic while there were only 51 females, and in 2021, males were 82 while females were 69, so overall the majority of patients were males with a percentage of 57.6% compared to 42.4% females as indicated Figures 1, 2. 

The notable increase in pediatric chest pain presentations from 2019 to 2021 can likely be attributed to a combination of psychological stress, increased health concerns, changes in lifestyle, and delayed healthcare access during the COVID-19 pandemic. While the study found that most cases were non-cardiac in origin, understanding these contributing factors is crucial for healthcare providers to address both the physical and psychological aspects of chest pain in children during and after the pandemic.

Common comorbidities

In the study, there was an increase in the percentage of patients with comorbidities from 32.6% in 2019 to 36.4% in 2021. Although the study does not provide detailed information on each specific comorbidity, typical comorbidities in pediatric patients presenting with chest pain may include: (i) Asthma and other respiratory conditions: These can contribute to chest pain related to shortness of breath or coughing; (ii) Obesity: Often linked with musculoskeletal pain or increased risk of acid reflux; (iii) Anxiety disorders: Psychological factors are known contributors to chest pain, particularly in non-cardiac cases; (iv) Gastrointestinal disorders: Conditions like gastroesophageal reflux disease (GERD) can cause chest pain, especially in children with poor dietary habits during lockdowns.

Table 1 displays that around 32.6% of the patients in 2019 had comorbidities, while in 2021, the percentage increased to approximately 36.4%. Echocardiography was normal in approximately 84% of cases in both years and ECG results were negative in the majority of patients with no significant difference between the two years (P-value = 0.236).

**Table 1:** Trends in patient comorbidities and diagnostic results: a comparison of 2019 and 2021

Year	2019	2021
Number of patients presenting to the clinic	1762	1532
Number of patients having chest pain (study sample)	132 (7.5%)	151 (9.9%)
Presence of comorbidity	With: 43 (32.6%)	With: 55 (36.4%)
	Without: 89 (67.4%)	Without: 96 (63.6%)

Regarding associated symptoms, patients mainly complained of palpitations, with 37.1% and 43% in 2019 and 2021, respectively. Shortness of breath was also common, with 37.9% in 2019 and 27.8% in 2021, but there was no significant difference in the presentation of associated symptoms between the two years, as shown in Table 2. The final diagnosis was categorized into two categories: cardiac and non-cardiac-related causes of chest pain. In 2019, 16 (12%) patients were diagnosed with cardiac-related causes compared to 23 (15%) patients in 2021. These results may be explained by the increase in parents' concerns about their children experiencing adverse reactions due to COVID-19 which played a role in affecting awareness and attitudes that led to an increase in the visits to the cardiology clinics.

**Table 2:** Comparison of symptom patterns and diagnostic outcomes for chest pain in 2019 and 2021

Association with other symptoms	Positive association 2019	Negative association 2019	Positive association 2021	Negative association 2021	P-value
Palpitations	49 (37.1%)	83 (62.9%)	65 (43%)	86 (57%)	0.311
Syncope	2 (1.5%)	130 (98.5%)	4 (2.6%)	147 (97.4%)	0.509
Stress	6 (4.5%)	126 (95.5%)	10 (6.6%)	141 (93.4%)	0.45
Shortness of breath (SOB)	50 (37.9%)	82 (62.1%)	42 (27.8%)	109(72.2%)	0.071
Dizziness	24 (18.2%)	108 (81.8%)	16 (10.6%)	135 (89.4%)	0.068

Table 3 reveals that there is no statistically significant difference between the two years in the final diagnosis.

**Table 3:** Consistency in final diagnoses: no significant change between 2019 and 2021

Year	2019	2021	P-value
Final diagnosis	Cardiac: 16 (12%)	Cardiac: 23 (15%)	0.45
	Non-cardiac: 116 (88%)	Non-cardiac: 128 (85%)	

Palpitations in cardiac vs. non-cardiac diagnoses

Palpitations were a common symptom among patients, reported by 37.1% in 2019 and 43% in 2021. While palpitations can occur in both cardiac and non-cardiac conditions, their presence often raises concern for potential cardiac causes. However, the study notes that a substantial number of cases with palpitations were associated with non-cardiac causes, such as anxiety or musculoskeletal issues, rather than serious heart conditions.

Cardiac vs. non-cardiac diagnoses

Cardiac Diagnoses

In 2019, 12% of patients had cardiac-related causes of chest pain, increasing slightly to 15% in 2021. Specific cardiac diagnoses in pediatric patients with chest pain typically include (i) Myocarditis: Inflammation of the heart muscle, which can present with chest pain, fatigue, and palpitations; (ii) Pericarditis: Inflammation of the sac surrounding the heart, often causing sharp chest pain; (iii) Arrhythmias: Abnormal heart rhythms, which may manifest as palpitations or chest discomfort.

Non-cardiac Diagnoses

The majority of chest pain cases were classified as non-cardiac in both years (88% in 2019 and 85% in 2021). Common non-cardiac diagnoses include (i) Musculoskeletal pain: The most common cause of non-cardiac chest pain in children, often linked to strain or injury; (ii) Respiratory issues: Conditions like asthma or infections that can lead to chest discomfort; (iii) Gastrointestinal causes: Acid reflux (GERD) or esophagitis, which can mimic chest pain; (iv) Psychological factors: Stress and anxiety, particularly elevated during the pandemic, are significant contributors to non-cardiac chest pain.

In summary, the study observed a significant increase in pediatric chest pain presentations in 2021 compared to 2019, highlighting an important trend during the COVID-19 pandemic. Despite this rise in cases, the majority of chest pain remained non-cardiac in origin, with common diagnoses including musculoskeletal and respiratory causes. Interestingly, only 4.6% of patients in 2021 were confirmed to have had COVID-19, and most of these cases were attributed to muscular causes rather than cardiac complications. Additionally, none of the patients who visited in 2021 received the COVID-19 vaccine at the time of their visit

Implications of the result

Increased Healthcare-Seeking Behavior

The increase in chest pain cases is likely influenced by heightened parental concern and anxiety during the pandemic rather than a surge in serious cardiac conditions. This suggests that the psychosocial impact of the pandemic may have played a significant role in driving more healthcare visits for benign conditions.

 *Low Incidence of COVID-19-Related Chest Pain*

The small percentage of patients with confirmed COVID-19 infections who presented with chest pain and the predominance of non-cardiac causes among them indicate that COVID-19 may not have been a major contributor to the increase in chest pain cases. This finding highlights the importance of distinguishing between pandemic-related anxiety and true medical emergencies in pediatric patients.

Stable Cardiac Diagnoses

The rate of cardiac diagnoses remained low and stable across both years, emphasizing the need for clinicians to focus on thorough evaluations to rule out serious cardiac issues while also addressing the psychological and musculoskeletal factors contributing to chest pain during the pandemic.

## Discussion

Key findings

Our study provides significant insights into the prevalence and characteristics of pediatric chest pain presentations. We observed a notable increase in the number of patients presenting with chest pain in 2021 compared to 2019. This trend is concerning and likely reflects heightened anxiety among patients and their parents following the COVID-19 pandemic. Interestingly, there were no significant differences in associated symptoms between the two years, indicating that the rise in chest pain cases may be influenced by factors beyond changes in symptom presentation. This underscores the need for ongoing research to explore potential underlying causes for this increase in 2021.

Despite the rise in cases, the proportion of patients diagnosed with cardiac-related causes of chest pain remained stable across both years. This finding suggests that the increase in chest pain presentations in 2021 did not correspond with a higher rate of serious cardiac diagnoses. This is consistent with a recent study, which indicated that in most cases of COVID-19-related cardiovascular symptoms, myocardial damage was due to the severity of the illness rather than direct viral injury to the myocardium [6]. Furthermore, only a small fraction of patients in 2021 had confirmed COVID-19 infections, with most cases attributed to muscular causes. This suggests that COVID-19 may not be a major contributor to the observed rise in chest pain, although further research is warranted. A systematic review and meta-analysis across all age groups also support that COVID-19 can cause a range of cardiovascular symptoms, including chest pain and arrhythmias [7].

An important observation from our study is that none of the patients who sought care in 2021 had received the COVID-19 vaccine, likely due to the vaccine’s later availability for the pediatric population. This highlights the need for continued efforts to encourage vaccination, which may help reduce the overall burden of chest pain. Supporting this, a recent study from Singapore confirmed the safety of COVID-19 vaccination in adolescents [8].

Comparison with existing literature

Our findings align with other research highlighting an increased focus on pediatric chest pain evaluations during the COVID-19 pandemic. Both studies found an increase in chest pain cases without a corresponding rise in serious cardiac conditions [9]. Both studies suggest that heightened attention to chest pain, driven by concerns related to COVID-19 and complications like multisystem inflammatory syndrome in children (MIS-C), led to more frequent evaluations. However, the majority of these cases were benign, with serious cardiac involvement remaining rare. Further research is needed to determine whether this trend continues post-pandemic and to assess the long-term impacts of increased diagnostic interventions during this period [10].

Additionally, the substantial rise in pediatric chest pain cases during the post-COVID-19 period highlights a swift return to healthcare-seeking behaviors, as evidenced by a shorter median time from onset to visit. Both our study and comparative analyses indicate that while case volumes surged, the rate of cardiac diagnoses remained low and stable. This trend underscores the importance of maintaining vigilance in screening for cardiac conditions to prevent missed or misdiagnoses during periods of heightened healthcare demand [11].

Implications for patient care

Our findings have significant implications for clinical practice. Despite increased presentations, the stable rate of serious cardiac diagnoses highlights the clear need for clinical guidelines addressing the psychosocial context of pediatric chest pain. These guidelines could involve thorough assessments considering psychosomatic factors to ensure a holistic evaluation of children. Additionally, implementing screening protocols that prioritize distinguishing between benign and serious conditions could help reduce parental anxiety and guide appropriate care. Our study emphasizes the ongoing need for education and support for healthcare providers in navigating the complexities of pediatric presentations in the post-pandemic landscape.

Future research directions

To gain a better understanding of the rise in pediatric chest pain cases, it is important for future studies to delve into specific psychosocial factors that contribute to increased anxiety and the tendency to seek healthcare. Valuable insights can be obtained through methodologies such as longitudinal studies, qualitative interviews, and surveys that assess levels of anxiety in parents and psychosomatic symptoms in children.

Furthermore, it will be crucial to investigate the long-term patterns of pediatric chest pain after the pandemic, including any potential changes in clinical outcomes and patterns of healthcare utilization. Research inquiries should center around whether this trend persists, what interventions could help alleviate the psychosocial effects, and how healthcare systems can adjust to these evolving needs.

Overall, our study provides valuable information about the prevalence and characteristics of patients who present with chest pain in a clinical setting. These findings may have important implications for patient care and highlight the need for ongoing research to identify potential underlying causes of chest pain and to develop effective interventions to reduce its burden.

Limitations 

Retrospective Design

This study relies on a retrospective design, utilizing data collected from medical records. This approach may lead to incomplete or inconsistent information, which can significantly impact the reliability of the findings. Incomplete data could obscure trends or associations, particularly concerning specific demographics or clinical outcomes. For example, missing entries related to patient history or treatment responses might skew the analysis, leading to inaccurate conclusions about the effectiveness of interventions across different groups.

Limited Generalizability

The research is conducted at a single institution, the University of Jordan Hospital in Amman, Jordan. This limitation raises questions about the generalizability of the findings to other settings or populations. The unique demographic and clinical characteristics of the hospital's patient population may not reflect broader trends, potentially introducing bias in the results. Moreover, variations in healthcare practices, patient behaviors, and socioeconomic factors across different regions could further limit the applicability of the study’s conclusions to other contexts.

Overall, these limitations suggest that the findings should be interpreted with caution, and further research is needed to validate the results across diverse populations and settings.

## Conclusions

Our research shows a notable rise in the number of children with chest pain over the two years studied. However, there were no significant differences in the characteristics of the patients, such as their demographics, associated symptoms, and underlying causes of chest pain between the two years. It is worth noting that psychological and social factors might be important in the increased awareness and concern about chest pain during the pandemic, rather than a direct impact of the disease itself. The findings suggest that the increase in cases may be due to changes in how people access healthcare, influenced by the pandemic. This emphasizes the importance of educating patients about chest pain concerns and distinguishing between benign and serious causes. Additionally, understanding the long-term trends in pediatric chest pain could inform healthcare policies, especially in terms of resource allocation and intervention strategies.

Future research should delve deeper into the underlying causes of chest pain, including potential effects of vaccination and psychological factors, to create effective interventions. In summary, our study underscores the need for ongoing assessment of pediatric healthcare practices in light of the changing landscape shaped by the COVID-19 pandemic.
